# Chlorogenic Acid and Cinnamaldehyde in Combination Inhibit Metastatic Traits and Induce Apoptosis via Akt Downregulation in Breast Cancer Cells

**DOI:** 10.3390/ijms25126417

**Published:** 2024-06-11

**Authors:** Yusuff Olayiwola, Lauren S. Gollahon

**Affiliations:** Department of Biological Sciences, Texas Tech University, Lubbock, TX 79407, USA; yolayiwo@ttu.edu

**Keywords:** phytochemicals, natural products, chlorogenic acid, cinnamaldehyde, breast cancer, metastasis

## Abstract

Most reported breast cancer-associated deaths are directly correlated with metastatic disease. Additionally, the primary goal of treating metastatic breast cancer is to prolong life. Thus, there remains the need for more effective and safer strategies to treat metastatic breast cancer. Recently, more attention has been given to natural products (or phytochemicals) as potential anticancer treatments. This study aimed to investigate the synergistic effects of the combination of the phytochemicals chlorogenic acid and cinnamaldehyde (CGA and CA) toward inhibiting metastasis. The hypothesis was that CGA and CA in combination decrease the metastatic potential of breast cancer cells by inhibiting their invasive and migratory abilities as well as the induction of apoptosis via the downregulation of the Akt, disrupting its signal transduction pathway. To test this, wound-healing and Transwell™ Matrigel™ assays were conducted to assess changes in the migration and invasion properties of the cells; apoptosis was analyzed by fluorescence microscopy for Annexin V/propidium iodide; and immunoblotting and FACSort were performed on markers for the epithelial-to-mesenchymal transition status. The results show that CGA and CA significantly downregulated Akt activation by inhibiting phosphorylation. Consequently, increased caspase 3 and decreased Bcl2-α levels were observed, and apoptosis was confirmed. The inhibition of metastatic behavior was demonstrated by the attenuation of N-cadherin, fibronectin, vimentin, and MMP-9 expressions with concomitant increased expressions of E-cadherin and EpCAM. In summary, the present study demonstrated that CGA and CA in combination downregulated Akt activation, inhibited the metastatic potential, and induced apoptosis in different breast cancer cell lines.

## 1. Introduction

Breast cancer is the most common type of cancer in women globally, accounting for about 25% of cancers, and is the leading cause of cancer-related mortality in women [1]. While studies have shown that early detection plays a major role in survivorship [1], once breast cancer becomes metastatic, survivorship decreases to less than 30% overall [2]. A recent study by Courtney et al. [2] showed that luminal A survivorship was 53.2%, and luminal B and triple-negative survivorship were both 18.5%. Thus, it is critical that new and more effective approaches to treating metastatic breast cancer be found, not just with the intention of prolonging life, but also to treat the disease.

One main reason for the lack of effective treatments against metastasis is its complexity. During metastasis, the primary tumor cells undergo genetic and phenotypic changes that facilitate intravasation from the primary site of development and extravasation to a distant site (for a review of the stages of metastasis, please see [3]). Our current knowledge describes it as a biological process that is characterized by the epithelial-to-mesenchymal transition (EMT), tumor invasion of the adjacent basement membrane and stroma, and migration through the vasculature, lymphatic vessels, trans-coelomic route, or even another path to secondary sites inside the body [4,5].

The EMT is a biological process that involves a highly coordinated set of changes, during which epithelial cells lose their characteristics as they transition into mesenchymal cells. During this transition, epithelial cells lose their adhesion properties, gaining an enhanced migratory capacity, and facilitating their invasion into the extracellular matrix. Ultimately, epithelial cell markers are downregulated with the concurrent upregulation of mesenchymal cell markers (for a current understanding of this process, please see Jayachandran et al.’s study (2021) [6]). Some of the key epithelial cell markers that are downregulated include E-cadherin, claudins, cytokeratin, and type IV collagen [7]. Concomitantly, mesenchymal cell markers like vimentin, fibroblast specific protein-1 (FSP), fibronectin, and N-cadherin become upregulated [7]. With respect to motility and membrane plasticity, there is a change in cytoskeletal components, and the strong cell–cell junctions (i.e., tight junctions, adherent junctions, and desmosomes) become lost, while filopodia and lamellipodia, critical to the migration capabilities of mesenchymal cells, increase [6].

While more effective and safer breast cancer treatment modalities are being developed for breast cancers that have not metastasized (see Wang and Wu’s study (2023) [8]), targeting the metastatic cancer cells is a major goal to reduce collateral damage both locally and systemically. However, our ability to effectively target metastatic breast cancer cells specifically is a herculean undertaking. Recently, more attention has been paid to phytochemical-based approaches to different diseases, including cancer [9,10].

Phytochemicals are biologically active secondary metabolites that display various pharmacological traits [9]. Also known as natural compounds, they are bioactive agents present in vegetables, fruits, legumes, spices, and teas [11]. They have been shown to demonstrate anticancer and therapeutic properties [11]. Phytochemicals include carotenoids, daidzein, curcumin, resveratrol, punicalagin, ellagic acid, genistein, and silibinin [9,11]. Phytochemicals have been shown to affect different molecular pathways that are responsible for the tumorigenicity of different breast cancer molecular subtypes both in vitro and in vivo [9,12,13,14,15]. There is a strong rationale for investigating this evolutionary link between plants and animals, especially given our dependency on plant-based food for normal health and physiology [9,10]. Furthermore, scientific inquiry is catching up to the intuitive medicinal knowledge of our ancestors with regard to the applications of phytochemicals in human health [16]. Combinations of different phytochemicals for treatments of cancer cells have been shown to produce a stronger biological impact than the individual components [17,18,19]. In our prior studies [18,20,21], it was shown that plant extracts within a mixture killed cancer cells with little or no cytotoxicity to normal cells. Thus, the idea was not to specifically target metastatic cancer cells but to exploit the co-evolutionary relationship of humans with natural products to “shower the cells” with phytochemicals. The cancer cells, too altered from normal cell function, become susceptible to changes in Akt signaling, resulting in CGA–CA-induced death.

AKT is important in the initiation of many pathways in both normal and tumor cells. As a “master regulator”, when phosphorylated, active AKT is shown to modulate at least 10 regulatory proteins [22]. Also known as PI3K/Akt/mTOR signaling, this pathway, in response to extracellular signals, promotes cell survival and growth [23]. Upon generation from phosphatidyl inositol 3,4-bisphosphate (PIP2) by the action of phosphoinositide 3-kinase (PI3K), PI 3,4,5-triphosphate (PIP3) recruits cytosolic Akt to the proximal region of the cell membrane where it is activated by phosphoinositide-dependent kinase 1 (PDK1). PI3K is activated by the binding of extracellular ligands to receptor tyrosine kinases (RTKs), and G-protein-coupled receptors (GPCRs) and by the RAS family of GTPases [24]. Thus, the Akt cascade is activated by various signals including RTKs, GPCRs, integrins, cytokine receptors, and B and T cell receptors. Activated Akt then phosphorylates and activates several other downstream proteins, including those residing in the cytoplasmic region of the plasma membrane, cytosol, and nucleus, thereby bringing about several biological effects, such as cell proliferation and survival. Several pathways under Akt regulation include mTOR, GSK3β, FOXO, and Mdm2-p53, among others [24]. There is also crosstalk with pathways such as MAPK, NF-kB, Wnt/β-catenin, and so on [25]. Relevant to this study is the relationship between AKT signaling and the EMT.

Chlorogenic acid and cinnamaldehyde are two important natural compounds that have been investigated for their anticancer effects on different molecular subtypes of breast cancer, including metastasis [18,26,27,28,29]. Chlorogenic acid (CGA) is a polyphenolic compound found in different plant sources such as apples, coffee, and carrots. Various studies have reported its anticancer properties [26,27,30], as well as its regulation of lipid and sugar metabolism (for a review, see [31,32]). Prior studies have reported that CGA demonstrates anticancer effects in colon cancer cells by increasing reactive oxygen species (ROS) generation and inhibiting proliferation [33]. Furthermore, CGA has been shown to induce apoptosis in lung cancer cells [34] (for a review of the potential therapeutic applications, please see [35]). Cinnamaldehyde (CA) is a phenylpropanoid, an α, β-unsaturated aldehyde, found in cinnamon tree bark and other plants of the genus Cinnamomum. The natural compound has been revealed to possess anticancer effects [28,29,36] as well as a plethora of pharmacological effects, as discussed in [7]. Prior studies have demonstrated the anticancer effects of CA on breast and other types of cancer cells, including ovarian [36] and non-small-cell lung cancer cells [37], by the disruption of the EMT via different pathways [38]. Thus, while the potential for the application of CA and CGA as anticancer treatment strategies becomes more relevant, there is still a need to elucidate their mechanisms of action with respect to cancer cell invasion and decreased metastatic traits.

Therefore, the aim of this study was to investigate the ability of chlorogenic acid and cinnamaldehyde combination (CGA–CA) to inhibit metastasis and initiate the intrinsic apoptotic pathway in the MDA-MB-231 and MCF-7 breast cancer cell lines. The underlying hypothesis was that CGA–CA in combination would inhibit Akt activation, thereby downregulating the Akt signaling pathway, and affecting downstream response proteins. Additionally, this Akt downregulation due to CGA–CA treatment would inhibit breast cancer cell invasion and migration and drive caspase-dependent apoptotic cell death. Thus, investigating the effects of CA–CGA on the phosphorylation status of Akt and its consequent effects on the EMT facilitates our understanding of the effects of phytochemicals on specific tumor-promoting pathways.

## 2. Results

### 2.1. CGA–CA Treatments Significantly Affected Breast Cancer Cell Proliferation

**Figure 1:**
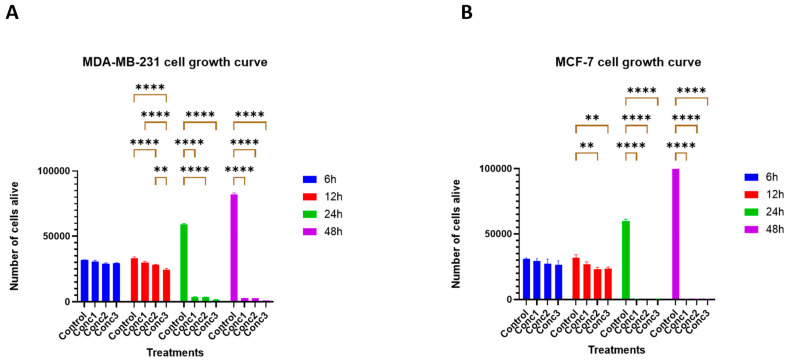
Anti-proliferative effects of CGA–CA combinations on (**A**) MDA MB-231 cells and (**B**) MCF-7 cells. Starting at 12 h, the CGA–CA combinations demonstrated significant anti-proliferative effects on both MDA-MB 231 and MCF-7 cells. Each bar represents the mean ± SEM of three biological replicates (*n* = 3). ** = *p* < 0.01, and **** = *p* < 0.0001, with *p*-value > 0.05 considered statistically nonsignificant (ns) and not indicated on the graph to reduce clutter.

The anti-proliferative capacity of the CGA–CA combinations in the MDA-MB-231 and MCF7 cells breast cancer cell lines was investigated by conducting cell growth curve analysis at different time points of 6, 12, 24, and 48 h. While the untreated cells continued to grow throughout the experiment, the proliferative ability of both cell lines was significantly impacted by the CGA–CA combinations. As shown in Figure 1, the antiproliferative potential of the CGA–CA mixtures was not observed at 6 h of treatment. However, at 12 h, a reduction in proliferation was observed for concentrations 2 and 3 in both cell lines. Interestingly, at 24 h of treatment, not only were the cells unable to proliferate, but there were also too few attached to use in further analyses. By 48 h of treatment, the cultures for both MDA-MB-231 and MCF7 cells were basically debris fields for all the concentrations of CGA–CA.

### 2.2. CGA–CA Treatments Caused Apoptotic Cell Death in Breast Cancer Cells

**Figure 2:**
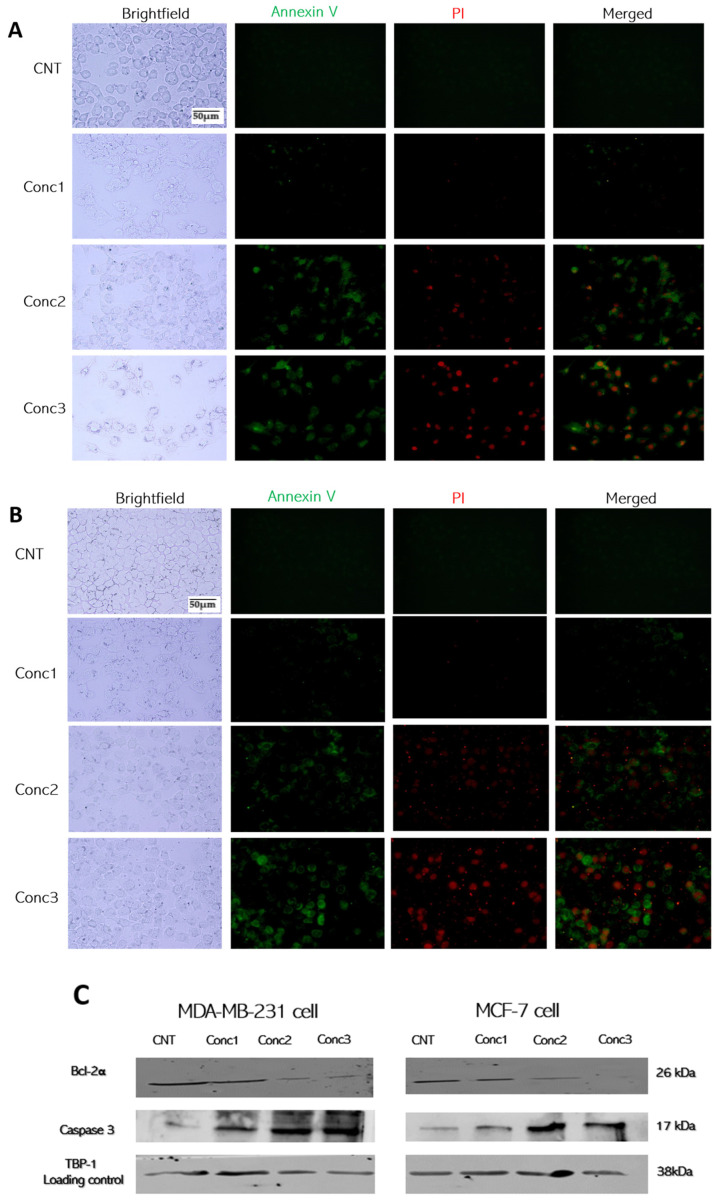

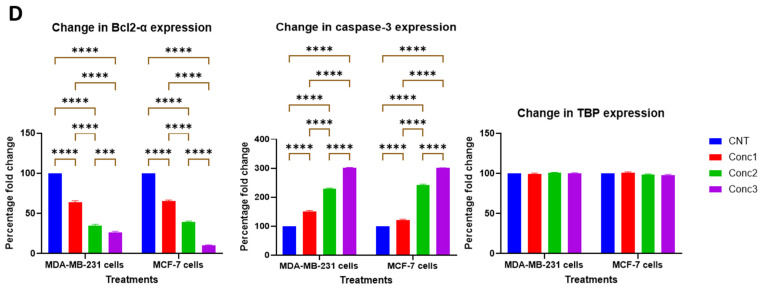
Apoptotic percentages in MDA-MB-231 and MCF-7 breast cancer cells induced by CGA–CA mixtures. Fluorescence microscopy analysis of annexin V and propidium iodide-stained (**A**) MDA-MB-231 and (**B**) MCF-7 cells. Representative fluorescence microscopy images of FITC-conjugated annexin V (green) and PI (red) double-fluorescence staining showing MDA-MB-231 and MCF-7 breast cancer cell apoptosis after treatment with three different concentrations of CGA–CA. All images processed under same conditions. Scale bar = 50 μm (**C**) Western blot showing the changes in expressions of caspase 3 and Bcl-2α in cells treated with varied concentrations of CGA–CA. TBP = loading control. (**D**) Quantitative representation of the expression levels of each of the proteins from (**C**). The results of three different biological replicates are expressed as means ± SEM. Image processing was performed using ImageJ software (version v1.54i). The statistical significance between the CGA–CA-treated groups and the control group for both cell lines was measured using one-way ANOVA followed by Tukey’s post hoc test for multiple comparisons. *** = *p* < 0.001, and **** = *p* < 0.0001. Statistically nonsignificant results are not included in the graphs to avoid clutter.

The induction of apoptosis by CGA–CA synergism in both MDA-MB-231 and MCF-7 cells was investigated in the present study. The ability of this phytochemical combination to promote breast cancer cell apoptotic cell death was examined using FITC-conjugated annexin V (the externalization of phosphatidylserine (PS) and a marker of early apoptosis) and propidium iodide (a DNA-intercalating dye and marker of later stages of apoptosis and necrosis). The untreated control cells showed almost no FITC-conjugated annexin V (no green fluorescence) signal, indicating non-apoptotic, live cells in both MDA-MB 231 and MCF-7 cells (Figure 2). In contrast, a small number of early apoptotic events were observed in both cell lines treated with concentration 1 of the CGA–CA mixture. At concentration 2 of CGA–CA, a number of annexin and PI signals were observed, indicating late-stage apoptosis. The concentration 3 CGA–CA treatments resulted in a stronger signal intensity and number for both annexin V and PI, as well as PI alone (indicating necrosis). A qualitative comparison of Figure 2A,B suggests that the MCF-7 cells were more sensitive to the CGA–CA treatments. An analysis of two key markers of apoptosis by Western blot is shown in Figure 2C. Caspase 3 is a key protein inducing the intrinsic apoptotic pathway [39,40]. In contrast, BLC-2α is an anti-apoptotic marker [39,40]. Western analysis revealed that the concentrations 2 and 3 CGA–CA treatments showed significant increases in caspase 3 and concurrent decreases in BCL-2 compared with the untreated controls.

### 2.3. CGA–CA Treatments Inhibited Breast Cancer Cell Migration

**Figure 3:**
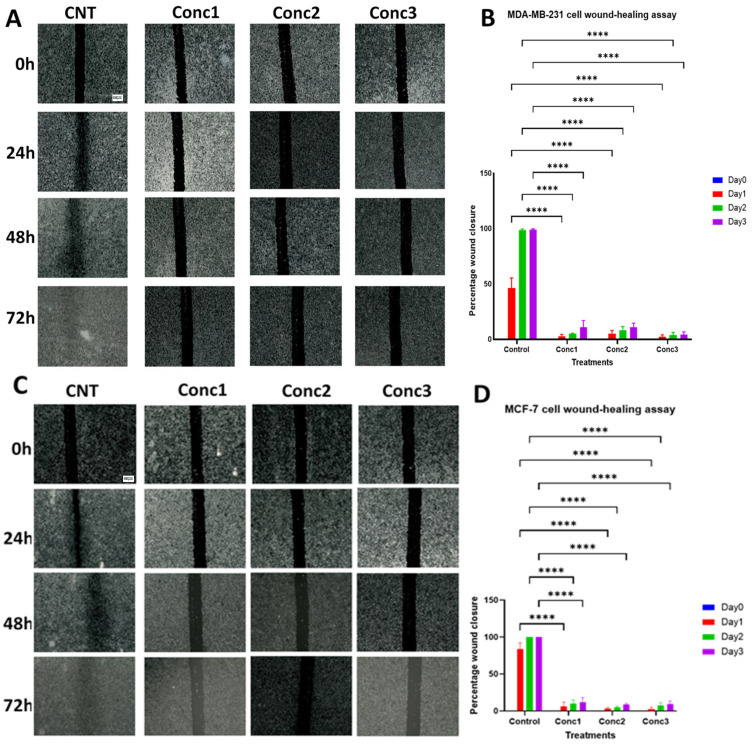
Wound-healing assay to assess anti-migration impact of CGA–CA on MDA-MB-231 and MCF-7 cells. (**A**,**C**) are representative photomicrograph images (scale bar = 100 μm; 4× mag) of wound-healing assays of MDA-MB-231 and MCF-7 cells, respectively, at 0 h, 24 h, 48 h, and 72 h. Images were processed with ImageJ software (version v1.54i). (**B**,**D**) are summary bar charts of the percentage of wound closure in both MDA-MB-231 and MCF-7 cells treated with three different doses of CGA–CA at 0 h, 24 h, 48 h, and 72 h, respectively. Three different biological replicates are expressed as means ± SEM of the percentage of wound closure. The statistical significance for the wound closure among the CGA–CA-treated cells in comparison with the untreated cells over time, for each cell line, was measured using one-way ANOVA, followed by Tukey’s post hoc test for multiple comparisons. **** = *p* < 0.0001. Statistically nonsignificant results are not indicated in the graphs to reduce clutter.

The capability of the phytochemicals to inhibit breast cancer cell migration was examined in this study by conducting a wound-healing assay using a novel device for creating straight and reproducible scratches in the cells [41]. MDA-MB-231 and MCF-7 cells were grown until confluence, and scratches/wounds were created in the cells. The images of the scratches were taken at different time points (0 h, 24 h, 48 h, and 72 h). The areas of the scratches were measured at each time point, and the percentage of wound closure was calculated. This was determined as the size of the wound closed at each time point relative to the original size of the scratches at time zero (0 h), expressed as a percentage (T_0_ − T_F_/T_0_ × 100%). Biological triplicates were carried out to ensure accurate results. The results show that the CGA–CA combinations completely shut down breast cancer cell migration (Figure 3). As expected, the untreated, control cells quickly migrated and closed the wound area within 48 h. All three CGA–CA combination concentrations prevented gap closure throughout the 72 h period of the assay for both cell lines. The quantification of the area of the wound at the different time points indicated that there was a statistically significant difference in the wound closure percentage of the treated cell groups for all three treatment concentrations compared with the untreated cells (Figure 3). The area and closure calculations were performed using the ImageJ software (version v1.54i), and all the parameters used for the analysis were kept equal for all images during the process. The anti-migratory ability of the phytochemicals was pronounced for concentrations 2 and 3 in both cancer cell lines.

### 2.4. CGA–CA Treatments Shut down the Invasive Ability of Breast Cancer Cells

**Figure 4:**
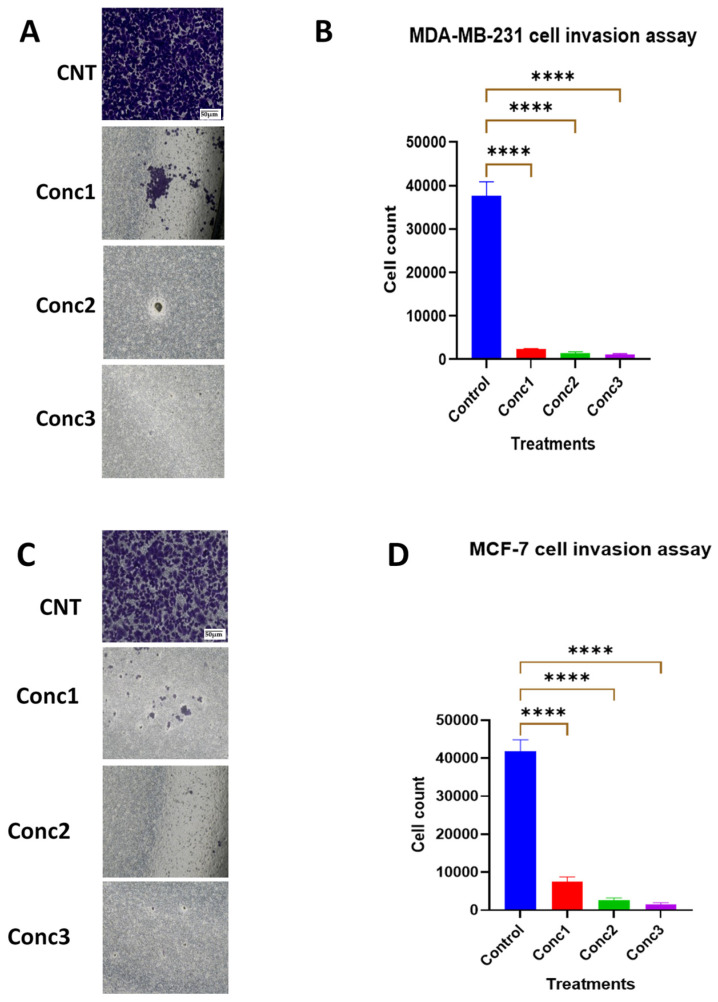
Transwell™ Matrigel™ invasion assay to assess anti-invasion capability of CA–CGA in MDA-MB-231 and MCF-7 breast cancer cell lines. (**A**) and (**C**) are representative photomicrographs of (scale bar = 50 μm, 10× mag) of MDA-MB-231 and MCF-7 breast cancer cells, respectively, via the Transwell™ Matrigel™ matrix. Cells were stained with crystal violet. (**B**,**D**) are bar graphs of the number of invasive cells in each group treated with three different doses of CGA–CA. Analysis of the invasive cells was carried out by acetic acid quantification method. The results of three different biological replicates are expressed as means ± SEM of the invasive cells. The statistical significance for the number of invading cells among the CGA–CA-treated groups in comparison with the control group for both cell lines was measured using one-way ANOVA, followed by Tukey’s post hoc test for multiple comparisons. **** = *p* < 0.0001. Statistically nonsignificant results are not included in the graphs to avoid clutter.

Cancer cell invasion of the extracellular matrix is a hallmark of tumor metastasis that precedes cell migration to distant tissues. The Transwell™ Matrigel™ cell migration assay was used to analyze the potential of the phytochemical mixture to inhibit breast cancer cell infiltration and penetration into the recapitulated basement membrane. Interestingly, all three concentrations of CGA–CA examined significantly inhibited the invasiveness of the cancer cells tested (Figure 4) compared with the controls. While the invasion suppressive effects of the phytochemical mixture appeared most pronounced for concentration 3 (Conc3), there was no statistically significant difference between the three different concentrations used. This result indicates that CGA–CA synergistically suppressed invasion in breast cancer cells in vitro.

### 2.5. CGA–CA Treatment Inhibited Akt Phosphorylation in Breast Cancer Cells

**Figure 5:**
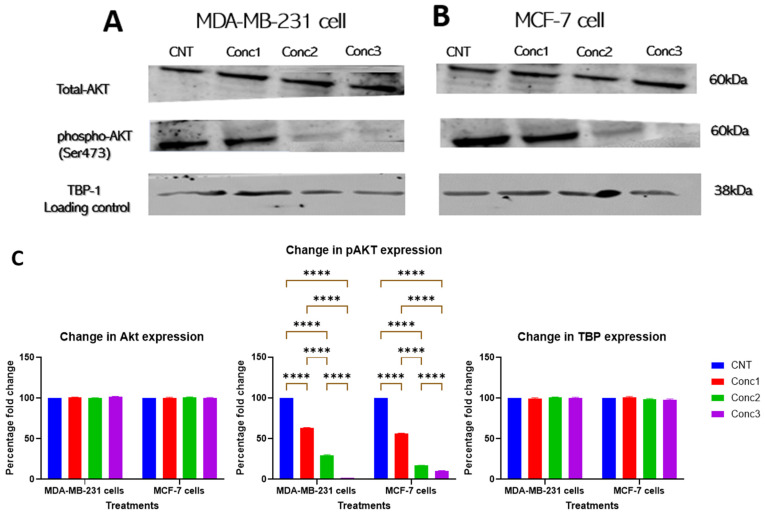
Akt activation was inhibited by CGA–CA in both MDA-MB-231 and MCF-7 cell lines. Western blotting shows the decreased levels of phosphorylated Akt in (**A**) MDA-MB-231 and (**B**) MCF-7 cells treated with CGA–CA. (**C**) Quantitative analysis of the expression levels of each of the proteins. TBP = internal loading control. The results of three different biological replicates are expressed as means ± SEM. The statistical significance between the groups for both cell lines was measured using one-way ANOVA, followed by Tukey’s post hoc test for multiple comparisons. **** = *p* < 0.0001. Statistically nonsignificant results are not included in the graphs to avoid clutter.

Akt is a protein kinase that is crucial to the regulation of several important signaling pathways in breast cancer, including migration and invasion [42]. Inhibiting the phosphorylation of Akt is important in suppressing cancer cells’ metastatic potential. Thus, the inhibition of Akt activation by CGA–CA treatment was assessed by Western blotting. The results indicate that the CGA–CA combinations inhibited Akt phosphorylation in both MDA-MB-231 and MCF-7 cells (Figure 5). There was a significant decrease in the expression levels of phosphorylated Akt (phospho-Akt) in the treatment groups compared with the untreated control cells. This was especially evident for concentrations 2 and 3 (Conc2 and Conc3). The suppression of Akt activation suggests that the compounds also affected various response/target proteins downstream of Akt signal transduction. Interestingly, the compounds did not affect the basal expression level of inactive, unphosphorylated Akt in either cell line.

### 2.6. CGA–CA Treatment Altered the Expression Levels of EMT Transition Markers

**Figure 6:**
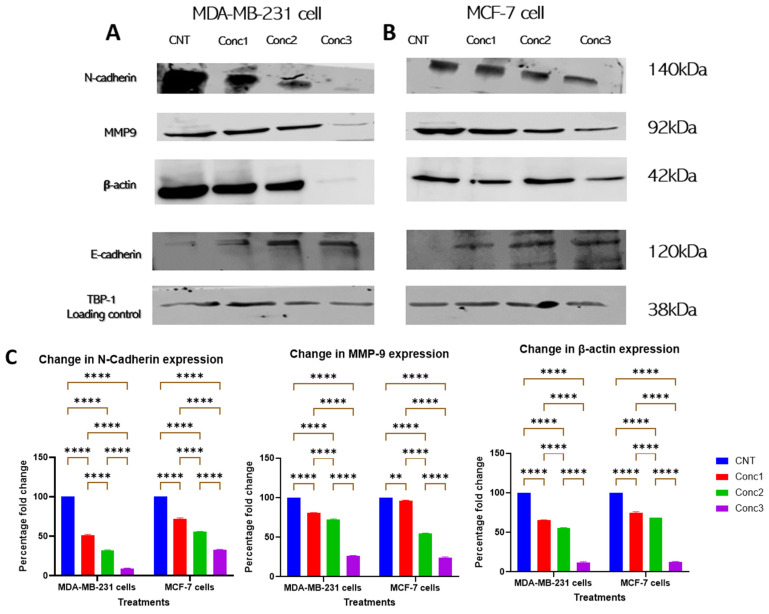

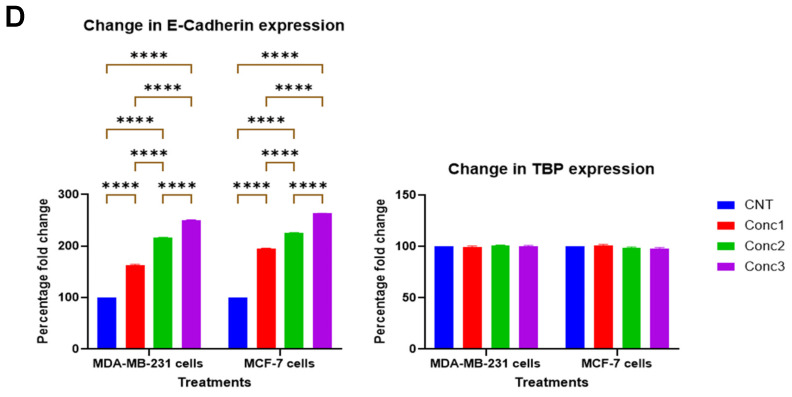
The impacts of CGA–CA treatments on expressions of proteins associated with epithelial-to-mesenchymal transition (EMT). Expression levels of EMT-associated proteins are shown in (**A**) MDA-MB-231 breast cancer cells and (**B**) MCF-7 breast cancer cells. (**C**) Quantitative results for the expression levels of each of the proteins associated with EMT. (**D**) Quantitative results for the expression levels of adhesion protein E-cadherin and loading control TBP. The results of three different biological replicates are expressed as means ± SEM. The statistical significance between the groups for both cell lines was measured using one-way ANOVA, followed by Tukey’s post hoc test for multiple comparisons. ** = *p* < 0.01, and **** = *p* < 0.0001. Statistically nonsignificant results are not included in the graphs to avoid clutter.

Figure 4 demonstrates that invasion was inhibited upon CGA–CA treatment, and Figure 5 shows the inhibition of Akt phosphorylation. To determine which downstream effectors in the Akt signaling pathway were affected, the expression levels of proteins associated with the epithelial-to-mesenchymal transition (EMT) were examined. The protein levels of N- and E-cadherin, MMP9, and β-actin analyzed by Western blotting are shown in Figure 6. These proteins are associated with cancer cell invasion and migration [43,44]. N-cadherin and MMP9 protein levels were downregulated by the CGA–CA treatments in a dose-dependent manner, with the greatest effects observed for concentration 3 (Conc3) in both cell lines (Figure 6). While the CGA–CA combinations suppressed the expression of β-actin in MDA-MB-231 cells, its expression level was not affected in MCF-7 cells. The expression level of E-cadherin, a marker of epithelial cell adhesion [43], was upregulated by the CGA–CA treatments in both breast cancer lines. The expression of E-cadherin, an epithelial cell phenotypic marker associated with adhesion, was upregulated in the CGA–CA treatments in a dose-dependent manner. This suggests a reduction in the metastatic phenotype in the MDA-MB-231 and MCF-7 cells and the restoration of non-motile epithelial characteristics.

**Figure 7:**
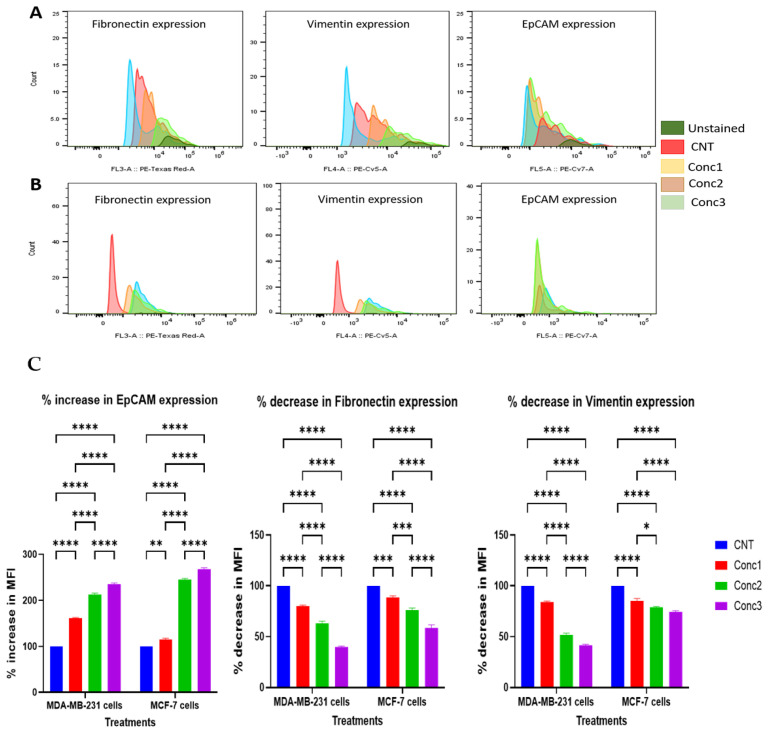
Summary results of EMT biomarkers fibronectin, vimentin, and EpCAM analyzed by flow cytometry. The flow cytometry data output was processed and analyzed with the FlowJo software (version V10.8.1). (**A**) MDA-MB-231 cells. (**B**) MCF-7 cells. Fibronectin and vimentin expression levels decreased in a dose-dependent manner with increasing CGA–CA treatment concentrations, while EpCAM expression increased in comparison with control breast cancer cells. (**C**) Quantitative results of the expressions of the proteins in both MDA-MB-231 and MCF-7 cells as a function of mean fluorescence intensity (MFI) of each cell expressing fibronectin, vimentin, and EpCAM. Expression levels were determined using FlowJo software (version v10.8.1) and fold changes in MFI, which corresponded to the expression levels of the proteins, calculated and expressed as percentages. The results of three different biological replicates are expressed as means ± SEM. The statistical significance of the percentage change in MFI among the CGA–CA-treated groups in comparison with the control group for both cell lines was measured using one-way ANOVA followed by Tukey’s post hoc test for multiple comparisons. * = *p* < 0.05, ** = *p* < 0.01, *** = *p* < 0.001, and **** = *p* < 0.0001. Statistically nonsignificant results are not indicated in the graphs to reduce clutter.

Additionally, the expression of the EMT biomarkers fibronectin, vimentin, and EpCAM were examined by flow cytometry (Figure 7). Fibronectin and vimentin are biomarkers associated with mesenchymal cells [45,46,47], while EpCAM expression is often observed in epithelial cells [48,49]. Fluorophore-conjugated primary antibodies were used to stain MDA-MB-231 and MCF-7 breast cancer cells. The expression levels of the target proteins were determined by FACsort using unstained cells as a control in the data analysis process. Interestingly, fibronectin and vimentin expressions were decreased in the treated groups in a dose-dependent manner compared with the untreated control cells of both cell types (Figure 7A,B). Conversely, EpCAM expression was upregulated in the treated breast cancer cells in a dose-dependent pattern (Figure 7A,B). Figure 7C is a summary of the quantitative results for the expressions of the proteins in both cell lines. The mean fluorescence intensity (MFI) of each cell expressing fibronectin, vimentin, and EpCAM was determined using the FlowJo software (version V10.8.1). The MFI is a measure of the intensity of the wavelength emitted by the fluorophore conjugated with the antibody, and, therefore, it is a measure of the relative abundance of the antigen or protein in the sample. Fold changes in the MFI, which corresponded to the expression levels of the proteins, were, therefore, calculated and expressed as percentages. The statistical significance of the percentage changes in the MFI among the CGA–CA-treated groups in comparison with the control group for both cell lines was measured using one-way ANOVA followed by Tukey’s post hoc test for multiple comparisons.

## 3. Discussion

Breast cancer is the most prevalent cancer in women globally, with an increasing mortality rate that appears to be unabated [50]. Metastasis is correlated with >90% of breast cancer-associated deaths [3,51,52]. Metastasis is a complex process involving a series of events that result in the formation of new tumors at a secondary site [3,4] through invasion and tumor dissemination. It is also a strong indicator of patient prognosis [53]. Thus, effectively treating metastasis with reduced side effects is the sin qua non of the treatment of breast cancer.

**Figure 8:**
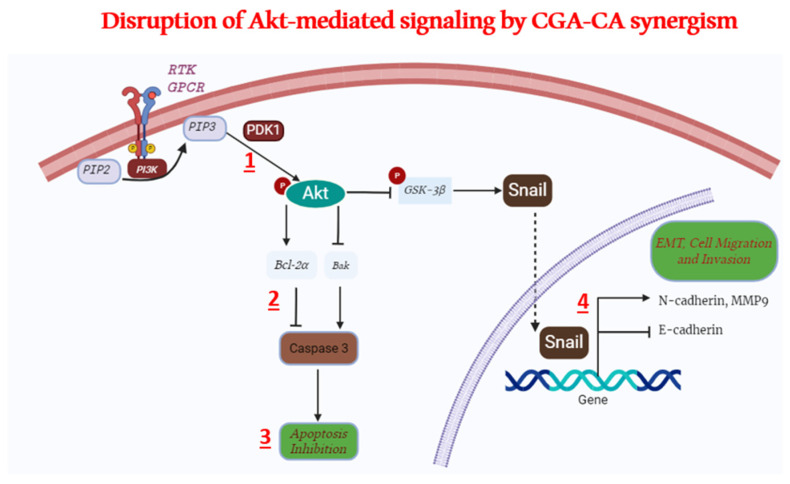
Effects of chlorogenic acid and cinnamaldehyde synergism on Akt-mediated signaling pathway downstream target proteins. Addition of CGA–CA inhibits phosphorylation of Akt (1) and inhibits expression of Bcl-2α (2), allowing induction of apoptosis via caspase 3 upregulation (3). Concurrently, inhibition of Akt phosphorylation affects N-cadherin and MMP9 expression (presumably by inhibiting SNAIL), causing upregulation of E-cadherin (4), and ultimately decreasing invasion and migration in breast cancer cells.

The aim of the current study was to investigate the capability of chlorogenic acid and cinnamaldehyde in combination to inhibit metastasis via the disruption of cell migration and invasion, as well as promoting apoptosis in breast cancer cells. Our previous work on CGA–CA alone and in combination showed that these compounds, together, killed breast cancer cells of different histotypes via mitochondrial dysfunction and did not significantly affect normal cells [18]. The results of our present study take this a step further and focus on the effects on the members of the Akt signal transduction pathway to inhibit metastatic behavior (Figure 8). The multi-step process of cancer cell metastasis is characterized mainly by the invasion of the surrounding basement membrane and adjacent cells and transmigration to near and distant tissues by the cancer cells [48]. Following genetic and epigenetic changes that drive the development of pre-metastatic cells and the formation of the pre-metastatic microenvironment, the first phase of metastasis is the local infiltration of tumor cells into the extracellular matrix (ECM) of adjacent tissue in a process called invasion [3,48]. Our results show that the CGA–CA combination was indeed able to arrest invasion and migration and induce apoptosis in both MDA-MB-231 and MCF-7 breast cancer cells. These findings are summarized in a potential mechanistic model in Figure 8.

Our results show that by inhibiting Akt activation, the activities of anti-apoptotic proteins such as Bcl-xL and Bcl2-α are downregulated. Presumably, pro-apoptotic proteins such as Bak and Bax are upregulated, resulting in the activation of the effector caspase 3. This results in apoptotic cell death in breast cancer cells. This hypothesis was confirmed, first, by the decrease in cell proliferation. A more focused investigation on apoptosis with annexin V/PI cell membrane integrity indicators revealed that there was a progression from early to late apoptosis and necrosis. The Western blot analysis of key apoptosis proteins showed that the level of Bcl2-α was significantly downregulated, while caspase 3 protein levels were increased in a dose-dependent manner by the natural compounds, and the cells were, indeed, observed to undergo apoptosis.

Migration is an intrinsic aspect of metastasis. After the infiltration of cancer cells into the stroma of adjacent tissues, the cancer cells can intravasate into the vascular or lymphatic circulation [43], and this is then followed by the extravasation and colonization of the secondary site [3]. Cellular movement requires organized cell–cell and cell–ECM adhesion, ECM remodeling, and cytoskeletal activity [3,54,55,56]. EMT is the molecular switch of polarized, non-motile epithelial cells, which normally exist as a collective sheet of cells with intact cell junctions connected to the basal lamina with their cell surface proteins, into motile, non-polarized mesenchymal-like cells [54]. The EMT confers an increased migratory capability and aggressiveness to breast carcinoma cells. The survivability of cancer cells is proportional to the ability of the cells to successfully metastasize, which, in turn, is directly linked to the migratory efficiency of the cells [57]. Therefore, targeting cancer cells’ migratory ability is central to destroying cancer cells’ ability to survive. Importantly, cancer cells metastasize to “search” for resources when the primary site is not favorable for their tumorigenicity. They move to newer sites to source nutrients and oxygen, according to the “seed and soil” theory of cancer metastasis [57]. Thus, inhibiting the motility of cancer cells is a therapeutic target.

Various agents are being tested to this end [5,14,57,58]. This current study showed that CGA and CA synergistically inhibit the migratory ability of breast cancer cell lines. The CGA–CA treatments, in a concentration-dependent manner, prevented the cancer cell lines from migrating across a gap compared with the untreated control cells in a wound-healing assay to assess cancer cell migration. The wound-healing assay conducted to assess the impact of CGA–CA on breast cancer cell migration revealed the ability of the phytochemicals to completely shut down the motility of both breast cancer cell types. Our CGA–CA treatment results are similar to findings of other phytochemical combinations’ impacts on breast and non-breast cancer cells, as previously reported in [37,59,60,61].

Invasion involves the remodeling and reconstruction of the ECM using proteolytic enzymes such as matrix metalloproteinases (MMPs) to create microchannels for cells to penetrate the stroma to reach vascular, lymphatic, or trans-coelomic routes [3,4,52]. Thus, successful invasion is central to metastasis. Therefore, disrupting invasion would be a strong therapeutic approach to stop the process of metastasis in breast cancer cells. In this study, CGA and CA in combination demonstrated the ability to prevent breast cancer cells from degrading extracellular matrix components in vitro. In the Transwell™ Matrigel™ invasion assay, CGA–CA significantly inhibited the ability of the MDA-MB 231 and MCF-7 breast cancer cell lines to digest the ECM compared with the untreated controls.

This was further validated by our immunoblotting and FACSort results. The expression level of MMP9 was significantly downregulated by the compounds. MMP9 is a proteolytic protein with essential roles in cancer metastasis and is an important target in breast cancer therapeutic research. It is a principal enzyme in the proteolytic degradation of the components of ECM [57]. Invasiveness is a characteristic of motile mesenchymal cells, and it is characterized by loss of cell–cell and cell–ECM interactions and the increased expression of various ECM-remodeling enzymes such as MMP9. Meanwhile, the reverse is true of non-motile epithelial cells. Compared with the controls, all the treatment concentrations decreased the expression of this enzyme.

N-cadherin is a cell surface protein, the increased expression of which corresponds to the loss of cell–cell adherence and increased cell motility in cancer cells [58]. E-cadherin and N-cadherin have antagonistic effects in the process of EMT, and the downregulation of E-cadherin with the concomitant upregulation of N-cadherin, called the cadherin switch, is considered critical for EMT in breast carcinoma [58]. Interestingly, CGA–CA in combination promoted the downregulation of N-cadherin in breast cancer cells. The combination of the phytochemicals, especially at Conc3, downregulated the expression of the mesenchymal cell marker compared with the untreated controls. The CGA–CA combination having downregulated the expression of N-cadherin suggests the loss of mesenchymal characteristics in both MDA-MB-231 and MCF-7 breast cancer cells.

Another interesting result was the apparent restoration of traits associated with differentiated epithelial cell phenotypes. Proteins associated with these traits that are usually lost during the EMT include E-cadherin and the epithelial cell adhesion molecule (EpCAM). EpCAM and E-cadherin are important cell–cell interaction molecules that are found on cell surfaces and play a role in the adherence of cells to one another and contribute to the non-motility of epithelial cells [48,49]. As epithelial cells switch to mesenchymal-like phenotypic characteristics in the EMT, the expression levels of these proteins are usually downregulated on the cell surfaces [45,56]. Our results showed that CGA–CA restored the expression of these proteins in the cancer cells exposed to the natural compounds. Our Western blot and flow cytometry analyses showed that the expressions of E-cadherin and EpCAM increased with the increase in the CGA–CA concentrations.

Vimentin is another biomarker of the EMT. It is usually found upregulated in mesenchymal and mesenchymal-like cells, such as transformed breast cells [45,62]. Vimentin is a type of intermediate filament that is part of cytoskeletal proteins, playing an important role in providing support and anchorage for various organelles in the cytosol of the cells [63]. It has been reported to play a role in cell motility and promoting the EMT by driving the cadherin switch in different types of cancer cells, including mammary tumors [45]. Vimentin expression is correlated with the survivability of breast cancer cells [62]. Therefore, downregulating the expression of this EMT molecule could negatively affect EMT progression in breast cancer cells. The expression level of the protein was significantly downregulated by CGA–CA treatment in a concentration-dependent manner compared with the untreated control breast cancer cells. The phytochemicals’ repression of the expression of this marker of the EMT explains, in part, the inhibited migratory capacity of the breast cancer cells in this study.

Fibronectin is a critical component of the ECM that mediates cell–ECM interactions necessary for cellular migration. It is a glycoprotein that binds to integrin receptor protein and other ECM components, such as collagen and laminin. Rick et al. [64] reported that it is synthesized and organized into scaffolds by cancer-associated fibroblasts, which drive the cancer cell invasion and migration processes [64]. An increased expression level of fibronectin is correlated with cancer cell migration and poor prognosis, and its pro-proliferative role in tumors has also been reported [59]; therefore, this ECM protein is a therapeutic target [57,64]. Inhibiting the expression level of fibronectin has been shown in previous studies to attenuate tumor growth and migration and reduce the survivability of neoplastic tissues [46,47]. The present study demonstrated that the CGA–CA combination represses the expression level of fibronectin and this, in part, may contribute to the anti-metastatic effect seen in breast cancer cells. CGA–CA’s downregulation of fibronectin increased in a dose-dependent manner in both the MDA-MB 231 and MCF-7 breast cancer cell lines.

### Study Limitations

Phytochemicals have been reported to modulate the Akt pathway in different types of cancer cells [13,60,61,65]. However, there are limitations to our study. Interestingly and importantly, the CGA–CA combination treatments investigated in the present study significantly inhibited Akt phosphorylation and its subsequent activation of downstream pathway proteins in the breast cancer cells in a dose-dependent manner. The compounds did not affect the expression level of basal unphosphorylated Akt in the cancer cells, suggesting that the dysregulated phosphorylation event alone was affected. The mechanism for this preferential disruption in phosphorylation is unknown, and further study is needed. However, it is possible that this inhibition could be achieved through the blockage of several cell surface receptors such as RTKs, GPCR, etc., that are upstream of Akt. The inhibition of Akt activation by CGA–CA could also be caused by the disruption of PI3K’s conversion of PIP3 from PIP2. Alternatively, the inhibition of the expression/activity of PDK-1, the enzyme responsible for phosphorylating and activating Akt, may also be disrupted [42,66]. These studies are ongoing. We also did not investigate the intermediate signaling protein SNAIL. An elucidation of its status may be informative for a better understanding of this key pathway.

It is still unclear how these phytochemicals enter breast cancer cells. This is an important consideration, and studies are underway. However, when these natural compounds enter the cytosol, they may act as either competitive inhibitors, binding to the Akt active site and thereby blocking ATP binding and its consequent activation [42] or behave as allosteric inhibitors by binding to the PH domain of the protein, thereby altering the Akt tertiary structure, which affects its function and makes Akt binding to other proteins impossible [42]. While the exact mechanism(s) of action of CGA–CA’s inhibition of Akt activation may not be known, our previous work [18,20,21], taken together with this study, suggests that phytochemicals affect multiple molecular targets in the cells to bring about their biological effects. One of these mechanisms involves the downregulation of Akt activation.

## 4. Material and Methods

### 4.1. Cell Lines and Cell Culture

The breast cancer cell lines used were MDA-MB-231 and MCF-7 cells. The MDA-MB-231 cell line was purchased from a human breast cancer cell line derived from the American Type Cell Collection (ATCC). MDA-MB-231 (ATCC, catalog# HT-26, ATCC, Gaithersberg, MD, USA) was derived from a pleural effusion and has a high metastatic capacity. It is a highly aggressive and poorly differentiated triple-negative breast cancer (TNBC) cell line, characteristically lacking both estrogen and progesterone receptors, as well as HER2 (human epidermal growth factor receptor 2) [37]. MCF-7 (ATCC, catalog# HT-22, ATCC, Gaithersberg, MD, USA) is a human breast cancer cell line positive for estrogen, progesterone, and glucocorticoid receptors. It is considered to have a low invasive potential and has been widely used for breast cancer research [38]. The breast cancer cell lines were cultured in high-glucose, Dulbecco’s modified Eagle’s medium (DMEM) (Gibco, Waltham, MA, USA) supplemented with 10% fetal bovine serum (FBS) (ATCC, Rockville, MD, USA) and 1% penicillin/streptomycin with 5% CO_2_ at 37 °C under humid conditions.

### 4.2. Cell Treatments

Chlorogenic acid (CGA) was purchased from Thermo Fisher (Thermo Fisher Scientific, Catalog# J60457.MD; Waltham, MA, USA) (≥95% purity) in powder form and was dissolved in PBS to a stock concentration of 300 μg/mL. Cinnamaldehyde (CA) was obtained from Thermo Fisher (Thermo Fisher Scientific, Catalog# A14689.36; Waltham, MA, USA) (98+% purity) in liquid form. The stock concentration was 7.9 mM. The CA was aliquoted, and the stock solutions were stored at room temperature according to the Manufacturer’s guidelines for further use. At the time of cell treatment, the stock solutions were added to the cell culture medium and then filtered through 0.22 μm pore size syringe filters (MilliporeSigma/Merck KGaA, Darmstadt, Germany). Previous work by our group determined the half-maximal inhibitory concentration (IC_50_) values of both CA and CGA in MDA-MB-231 and MCF-7 cells [18]. For CA, the concentrations were determined to be 42.5 µM and 35 µM, respectively [18]. The IC_50_ values of CGA in MDA-MB-231 and MCF-7 cells were 225 µg/mL and 250 µg/mL, respectively [18]. While these proved to have significant anticancer properties with no significant cytotoxicity, we were interested in determining whether a synergistic effect would remain efficacious at even lower doses of the phytochemicals. Based on these results [18], mixtures of CGA–CA at lower doses were combined and used in this study. Three different concentrations of each of the phytochemicals at ~25%, 45%, and 65%, and ~10%, 20%, and 40%, were combined and tested in the present study, as shown in Table 1. The working concentrations were made by diluting the stock concentrations of the phytochemicals with the DMEM culture medium. Three different doses of CGA–CA combinations were administered to the MDA-MB 231 and MCF-7 cell lines in this study to assess the impact of the synergism of the two phytochemical agents.

### 4.3. Cell Growth Curves Determining Anti-Proliferative Potential of Each CGA–CA Concentration

Log-phase MDA-MB-231 and MCF-7 cells were plated at 40,000 cells/well into 96-well plates, allowed to acclimate overnight, and treated with a 1:1 CGA–CA mixture at the different concentrations listed in Table 1, or they were left untreated. The cells were harvested at 6 h, 12 h, 24 h, and 48 h. At the end of each experimental time point, the cells were gently washed with phosphate-buffered saline (PBS) to remove dead cells and debris, and the attached cells were fixed with ice-cold absolute methanol. Following fixation, the cells were stained with 0.5% crystal violet staining solution for 30 min. Crystal violet dye binds to proteins and DNA and is used to quantify the cellular protein and DNA levels, and the amount of bound crystal violet dye is directly proportional to the cell number in the well [67]. After aspirating the staining solution, the plates were gently washed to remove excess unbound stain and air-dried at room temperature [67]. For quantification, 200 μL of 33% acetic acid was added into each well to solubilize the dye, and the wells were incubated for an hour on a plate shaker. The optical density was measured using a Bio-Tek Synergy H1 Microplate Reader H1M (Bio-Tek; Winooski, VT, USA) at a wavelength of 590 nm [67]. Concurrently, a standard curve was generated to extrapolate the actual number of cells from the absorbance obtained from each well. In total, 1000, 2000, 5000, 10,000, 20,000, and 40,000 cells for both MDA-MB-231 and MCF-7 were seeded into 96-well plates and allowed to attach to the bottoms of the well plates overnight. As described above, the cells were washed with PBS, fixed with ice-cold methanol, stained with crystal violet, and solubilized with acetic acid, as above. Following this, the absorbance was measured at a wavelength of 590 nm.

### 4.4. Annexin V Detection of Apoptosis

The annexin V apoptosis detection kit buffer (Invitrogen, Catalog# 88.8005-74, Waltham, MA, USA) was used for determining apoptosis. Briefly, log-phase MDA-MB-231 and MCF-7 cells were seeded at 30,000 cells/well in removable 8-well chamber slides (ibidi USA, Inc., Fitchburg, WI, USA). After overnight incubation for acclimatization, the plated cells were gently washed with PBS, and a fresh growth medium was added. The three CGA–CA treatment concentrations were added, and the cells were incubated for 9 h, after which the cells were gently washed twice with 100 μL of 1× binding buffer. Thereafter, the cells in the chamber slides were stained with FITC-conjugated annexin V and propidium iodide (PI) following the manufacturer’s instructions. The staining solution was prepared by adding 5 μL of FITC-conjugated annexin V and 5 μL of PI to 100 μL of 1× binding buffer and vortexed thoroughly. The staining solution was then carefully added to the attached cells in each of the chamber slides. The cells were then incubated at room temperature in the dark for 5 min. Next, the stain was removed, and the slides were air-dried at room temperature. The silicon seal and the plastic chamber on top of each slide were carefully removed, and the cells were evaluated using an Olympus BX51 microscope (Olympus, Center Valley, PA, USA) at 40× using FITC (Ex: 498 nm; Em: 517 nm) and PI (Ex: 493 nm; Em: 636 nm) filter cubes. All settings on the microscope were kept constant for all the samples. The images were processed and analyzed using ImageJ software (version v1.54i). The brightness/contrast and other adjustment parameters were kept constant for all the samples.

### 4.5. Wound-Healing Assay to Assess Anti-Migration Potential of CGA–CA Combinations

MDA-MB-231 and MCF-7 cells were seeded in 12-well plates at a density of 200,000 and 300,000 cells/well, respectively, in 2 mL of DMEM growth medium and allowed to reach 90–100% confluence at 5% CO_2_ and 37 °C, with humidity. The cells were synchronized for the cell cycle using a double-thymidine block, as described in Wolpert and Gollahon’s study (2024) [41]. At this point, a novel cell culture-based wound-healing platform, designed to produce accurate and reproducible straight-line gaps, was used to generate a score across each well [41]. The cells were then carefully washed with PBS to remove floating cells and debris. A fresh culture medium, with only 1% FBS, was carefully added along with the different concentrations of CGA–CA mixtures to each plate, or the cells were left untreated. FBS of 1% was used to maintain viability and halt cell proliferation. Thus, any wound closure observed would be due to cell migration and not cell proliferation. The created scratches in the well plates were inspected using light microscopy, and initial images of the scratches were captured with an EVOS XL Core imaging system (Thermo Fisher Scientific; Catalog# AMEX1000; Waltham, MA, USA) to measure the area of the gap generated at time zero (0 h). The cells were then returned to the incubator. Images of each of the scratches in the well plates were obtained at the 24 h, 48 h, and 72 h time points to measure the areas of the gaps and determine the rate or percentage of wound closure, which corresponded to the measurements of the migration rate. The images were processed and analyzed using ImageJ software (version v1.54i). The brightness/contrast and other adjustment parameters were kept constant for all the samples.

### 4.6. Transwell™ Matrigel™ Invasion Assay to Analyze Anti-Invasiveness of CGA–CA Treatment

The BD Matrigel™ matrix (BD Biosciences, Catalog# 356234; Franklin Lakes, NJ, USA) at a 10 mg/mL stock concentration was used to generate a basement membrane in the present study. The Matrigel™ matrix was thawed on ice in a 4 °C walk-in cold room for about 2 h, during which time the vial was swirled to ensure that the material was evenly dispersed prior to aliquoting. The Matrigel™ matrix was handled using a sterile technique and kept on ice throughout the experiment. An amount of 100 μL of the 200 μg/mL Matrigel™ final concentration was prepared from the stock by using FBS-free DMEM culture medium as a diluent, and the resulting coating solution was thoroughly mixed by gently swirling and then placed on ice. The coating solution was gently pipetted into the middle of the cell culture Transwell™ insert (Corning Inc., Product# 3422; Kennebunk, ME, USA) in each well of the 24-well plates and dispersed to cover the surfaces of the inserts. The plates were incubated to coat the membrane in each Transwell™ insert to generate a basement membrane in the in vitro system at 37 °C for 2 h. All the pipettes, syringes, and containers that were used with the Matrigel™ matrix were chilled before use. Just prior to use, the remaining coating solution that did not form a gel in the Transwell™ inserts was carefully removed without disturbing the layer of the Matrigel™ matrix on the membrane, and the coated invasion chambers were then ready for use without letting the Matrigel™ matrix layer dry out.

Next, 100,000 cells/500 μL of FBS-free DMEM culture medium were carefully seeded into the invasion chamber inside each well of the 12-well plate, while 1 mL of a complete growth medium was carefully added to the bottom of the wells of the plate, making contact with the membrane. The complete medium served as a chemoattractant to promote the invasion of the cells from the upper Transwell™ insert. The completed set-up was placed in an incubator for about 30 min to let the cells settle, after which the different concentrations of CGA–CA mixtures were added, or the cells were left untreated. The invasion assay set-up was left overnight to incubate at 37 °C and 5% CO_2_ with humidity. To terminate the experiment and determine the cell invasion into the Matrigel™ matrix and through the membrane pores to the bottom of the wells, the culture medium in the Transwell™ inserts and bottom wells of the plates were removed and gently rinsed with PBS. A cotton swab moistened with PBS was used to carefully remove the non-invading cells by holding the inserts carefully with forceps. Next, the cells were fixed with ice-cold absolute methanol. The fixed cells were stained with 0.5% crystal violet for 30 min. After removing the excess staining solution, the plates and Transwell™ inserts were gently washed by dipping them into distilled water several times and air-dried at room temperature [67]. The Transwell™ inserts were gently placed on microscope slides, and images of the invading cells were captured using an EVOS XL Core imaging system (Thermo Fisher Scientific; Catalog# AMEX1000; Waltham, MA, USA). For quantitative results, the fixed and stained cells from each well with the inserts were eluted with 300 μL of 33% acetic acid by incubation for an hour on an orbital shaker at room temperature. After elution, 100 μL of each solution was transferred into a 96-well plate to read the absorbance with a Bio-Tek Synergy H1 Microplate Reader H1M (Bio-Tek; Winooski, VT, USA) at a wavelength of 590 nm [67].

Concurrently, a standard curve was generated to extrapolate the actual number of invading cells from the absorbance obtained from each well. In total, 1000, 2000, 5000, 10,000, 20,000, and 40,000 of both log-phase MDA-MB-231 and MCF-7 cells were seeded into 96-well plates and allowed to attach to the bottoms of the well plates overnight without letting them double in number. As described above, the cells were washed with PBS, fixed with ice-cold methanol, and stained with crystal violet staining solution, with the excess stain washed off with water, and then solubilized with acetic acid. Then, the absorbance was measured at a wavelength of 590 nm.

### 4.7. Protein Extraction and Quantification

Protein extraction and quantification were performed as previously reported in [68]. Briefly, the Pierce RIPA lysis buffer (Lot# XG348655, Thermo Scientific, Waltham, MA, USA) was used for lysis and protein extraction from each cell line. A Halt protease–phosphatase inhibitor (100×) and 0.5 M EDTA (catalog# 78420, 78420, 87886; Thermo Fisher Scientific, Waltham, MA, USA) cocktail was added to the lysis buffer. The inhibitor cocktail, containing a spectrum of protease inhibitors and phosphatase inhibitors, protects proteins during extraction or lysate preparation from cells. An amount of 5 microliters of 100× protease, phosphatase, and EDTA was added to every 500 µL of lysis buffer to make a final concentration of 1×.

After 24 h of the MDA-MB-231 and MCF-7 cells’ treatment with different concentrations of CGA–CA combinations in 100 mm Petri dishes, the culture medium was removed, and the cells were gently washed with PBS. An amount of 1000 µL of ice-cold lysis buffer was added, and the Petri dishes were incubated at −80 °C for 3 min, after which the Petri dishes were put on a rocker for 15 min at 4 °C to enhance cell detachment from the Petri dishes and cell lysis. The cells were then collected in microcentrifuge tubes using an ice-cold plastic cell scrapper. Next, the microcentrifuge tubes were put in a laboratory shaker (Barnstead Thermolyne LabQuake Shaker, Marshall Scientific, Hampton, NH, USA) for 30 min at 4 °C for cell lysis. The microcentrifuge tubes were then spun for 15 min at 4 °C at 12,000 rpm. Each supernatant was collected as a whole cell lysate of the protein extract. Protein quantification was then carried out using the Pierce BCA Protein Assay Kit (Catalog#: 23225; Thermo Fisher, Waltham, MA, USA). The BCA protein assay was used to determine the protein concentrations in the whole cell lysates of the MDA-MB-231 and MCF-7 cells for treatment and groups of cells according to the manufacturer’s protocol. The reference standard protein (Pierce™ Bovine Serum Albumin Standard) concentrations were made as per the manufacturer’s instructions (Catalog#: 23209; Thermo Scientific, Waltham, MA, USA). A total of 200 µL of the protein analysis solution was added to 25 µL of the standard and sample proteins in triplicate. The absorbance was measured at 562 nm with a Bio-Tek Synergy H1 Microplate Reader H1M (Bio-Tek; Winooski, VT, USA).

### 4.8. SDS–Polyacrylamide Gel Electrophoresis Sample Preparation

A volume corresponding to 35 µg of protein from each sample of the whole cell lysate of the MDA-MB-231 and MCF7 cells was mixed with an equal volume of 2× Laemmli loading dye, and RIPA lysis buffer was used to fill the remaining volume to 40 µL. The samples were then denatured by boiling in a heat block at 95 °C for 5 min and centrifuged at 16,000 rpm in a microcentrifuge for 1 min.

Following this step, the samples were loaded into each well of an SDS–polyacrylamide gel. This hand-cast SDS-PAGE gel contained a 4% stacking gel and 8–10% gradient resolving gel. PageRuler™ Prestained Protein Ladder (Invitrogen, Catalog# 26617; Waltham, MA, USA) was used as a molecular weight marker. The PAGE was run at 120 V until the 10 kDa protein reached the bottom of the gel. Next, the resolved protein was transferred from the gel to a nitrocellulose membrane (Thermo Scientific, Lot# XE3443891, Waltham, MA, USA). The transfer was performed in an ice bath with 1× transfer buffer (25 mM Tris; 192 mM glycine) at 100 V for 90 min. After transfer, the efficiency of the transfer was examined using the Ponceau S solution (Thermo Scientific, Catalog# A40000279, Waltham, MA, USA).

### 4.9. Immunoblotting

Nitrocellulose membranes were blocked with 5% bovine serum albumin (BSA) blocking buffer made in 1× TBS-Tween buffer (Trizma HCl, NaCl, ultra-pure water, Tween 20) overnight. Following blocking, the membranes were incubated with primary antibodies at a 1:2000 dilution for 2 h at room temperature. The following primary antibodies were used: a rabbit Akt polyclonal antibody (Rockland, Catalog# 200-401-N98; Limerick, PA, USA), a rabbit phospho-Akt monoclonal antibody in the Ser473 position (Invitrogen, Catalog# 44-621G; Waltham, MA, USA), a rabbit MMP-9 monoclonal antibody (Product#13667, Cell Signaling Technology, Danvers, MA, USA), a rabbit N-cadherin monoclonal antibody (Product# 13116, Cell Signaling Technology, Danvers, MA, USA), an E-cadherin monoclonal antibody (Product# 3195, Cell Signaling Technology, Danvers, MA, USA), a rabbit β-actin monoclonal antibody (Product#4970, Cell Signaling Technology, Danvers, MA, USA), and a rabbit caspase 3 monoclonal antibody (Product# 4970, Bioss Antibodies, Woburn, MA, USA), and a rabbit TATA box-binding protein (TBP) polyclonal antibody (Invitrogen, Catalog# PA5-85773; Waltham, MA, USA) was used as the loading control. After the primary antibody incubation, the membranes were rinsed 3 times with 1× TBST wash buffer for 10 min each and then incubated with a goat anti-Rabbit IgG HRP-conjugated (Product# 7074, Cell Signaling Technology) secondary antibody for 90 min. Next, the membranes were rinsed 3 times with 1× TBST wash buffer for 10 min each. The membranes were then incubated with an ECL chemiluminescent substrate (Thermo Scientific, Catalog# 34577, Waltham, MA, USA) at room temperature for 5 min and visualized using the LiCor Odessey Imaging system (Odyssey^®^ Fc) by LI-COR Biosciences (Lincoln, NE, USA). The internal software was used to analyze the signals for statistical comparison.

### 4.10. Protein Expression Analysis by Flow Cytometry

The expression of markers related to the epithelial-to-mesenchymal transition (EMT) in MDA-MB-231 and MCF-7 cells was confirmed by flow cytometry. MDA-MB-231 and MCF-7 cells were treated with the CGA–CA mixtures at different concentrations for 24 h, after which the cells were gently washed with PBS and harvested with a trypsin/EDTA solution, and the cell suspensions were then prepared with PBS. The cell concentration was ~2 million cells per 50 μL of FACS buffer (1× Ca^2+^/Mg^2+^-free PBS). An amount of 50 μL of the cells was pipetted into flow tubes for staining. The samples were mixed by gently vortexing them and incubated for 10–15 min at 4 °C prior to the addition of the antibody cocktail, with a 50 μL total volume per tube. Cell staining was carried out by incubating the cells overnight with a combination of fluorophore-conjugated primary antibodies, which were Alexa flour 647-conjugated vimentin (Invitrogen, Waltham, MA, USA), Ugl-Y3-conjugated fibronectin (Invitrogen, Waltham, MA, USA), and PE-cyanine7-conjugated epithelial cell adhesion molecule (EpCAM). A volume of 0.2 μL of each antibody was carefully pipetted into 50 μL of FACS buffer, mixed thoroughly, and transferred to the single-cell suspensions for incubation overnight in the dark at 4 °C. Next, the cell suspensions were washed with FACS buffer and centrifuged, the supernatants were carefully removed, and the pellets were resuspended in 400 μL of FACS buffer. The relative expression levels of the proteins were measured by a BioSino ZS-AE7S flow cytometer (BioSino Biotechnology and Science Inc., Beijing, China), and the FlowJo software (version v10.8.1) was used for result analysis.

### 4.11. Statistical Analysis

The statistical analysis of the data was performed using the GraphPad Prism 9 software (GraphPad Software Inc., San Diego, CA, USA). For all analyses, at least three different biological replicates and three technical replicates were performed for each experiment to ensure the accuracy of the results expressed as means ± standard deviation (SD). The statistical significance between all the treatment groups and control groups of the cells was determined with analysis of variance (ANOVA). Based on the independent variables, one-way or two-way ANOVA was performed. Tukey’s post hoc test was employed for multiple comparisons between group means. *p* < 0.05 was considered to indicate a statistically significant difference. For all figures, significance is represented by the following: Alternatively, if all most of the comparisons are significant, then just non-significant (ns) exceptions are discussed.

## 5. Conclusions

The inhibition of Akt activation has several biological consequences due to its importance as a regulator for a multitude of signaling cascades in cells. The inhibition of the PI3K/Akt pathway is correlated with the direct and indirect inhibition of several pathways implicated in cancer cells, such as the MAPK [69], mTOR [70], GSK3β [71], FOXO, NF-kB [72], Wnt/β-catenin [73], and Mdm2-p53 [74] pathways. The fact that the compounds under investigation significantly downregulated the Akt pathway by the inhibition of Akt phosphorylation offers one explanation for why breast cancer cells in this study committed to apoptosis and were not able to undergo invasion and migration in comparison with untreated control cells. This study further confirmed the central role of the Akt pathway in carcinogenesis and cancer survival and the fact that Akt signaling is, indeed, a cancer therapeutic target. Taken together, the present data indicate that the chlorogenic acid and cinnamaldehyde synergism shut down PI3K/Akt pathway signaling and suppressed invasion and migration while promoting apoptotic cell death in MDA-MB-231 and MCF-7 breast cancer cells. The results show that the natural compound combination induced the reversal of the cadherin switch and promoted the mesenchymal-to-epithelial transition (MET) in the breast cancer cells. With these interesting results, further investigation will be needed to establish the capabilities of the phytochemicals in an experimental animal model of breast cancer to better gauge the anticancer effects of this CGA–CA combination on breast cancer progression when orally administered.

## Figures and Tables

**Table 1:** Dosage combinations of CGA and CA.

	MDA-MB 231 Cells		MCF-7 Cells	
	CGA	CA	CGA	CA
Conc1	25 µg/mL	10 µM	25 µg/mL	10 µM
Conc2	50 µg/mL	20 µM	50 µg/mL	15 µM
Conc3	100 µg/mL	30 µM	100 µg/mL	20 µM

## Data Availability

Data is available upon reasonable request to the corresponding author.
